# Absence of genetic structure in *Baylisascaris schroederi* populations, a giant panda parasite, determined by mitochondrial sequencing

**DOI:** 10.1186/s13071-014-0606-3

**Published:** 2014-12-23

**Authors:** Yue Xie, Xuan Zhou, Zhihe Zhang, Chengdong Wang, Yun Sun, Tianyu Liu, Xiaobin Gu, Tao Wang, Xuerong Peng, Guangyou Yang

**Affiliations:** Department of Parasitology, College of Veterinary Medicine, Sichuan Agricultural University, Ya’an, 625014 China; Centre for Animal Diseases Control and Prevention, Dachuan Animal Husbandry Bureau, Dazhou, 623000 China; Chengdu Research Base of Giant Panda Breeding, Chengdu, 610081 China; Department of Chemistry, College of Life and Basic Science, Sichuan Agricultural University, Ya’an, 625014 China

**Keywords:** *Ailuropoda melanoleuca*, Roundworm, Genetic diversity, Mitochondrial *atp6*, Mitochondrial *cox1*

## Abstract

**Background:**

Infection with the parasitic nematode, *Baylisascaris schroederi* (Ascaridida: Nematoda), is one of the most important causes of death in giant pandas, and was responsible for half of deaths between 2001 and 2005. Mitochondrial (mt) DNA sequences of parasites can unveil their genetic diversity and depict their likely dynamic evolution and therefore may provide insights into parasite survival and responses to host changes, as well as parasite control.

**Methods:**

Based on previous studies, the present study further annotated the genetic variability and structure of *B. schroederi* populations by combining two different mtDNA markers, ATPase subunit 6 (*atp6*) and cytochrome c oxidase subunit I (*cox1*). Both sequences were completely amplified and genetically analyzed among 57 *B. schroederi* isolates, which were individually collected from ten geographical regions located in three important giant panda habitats in China (Minshan, Qionglai and Qinling mountain ranges).

**Results:**

For the DNA dataset, we identified 20 haplotypes of *atp6*, 24 haplotypes of *cox1*, and 39 haplotypes of *atp6* + *cox1*. Further haplotype network and phylogenetic analyses demonstrated that *B. schroederi* populations were predominantly driven by three common haplotypes, *atp6* A1, *cox1* C10, and *atp6* + *cox1* H11. However, due to low rates of gene differentiation between the three populations, both the *atp6* and *cox1* genes appeared not to be significantly associated with geographical divisions. In addition, high gene flow was detected among the *B. schroederi* populations, consistent with previous studies, suggesting that this parasite may be essentially homogenous across endemic areas. Finally, neutrality tests and mismatch analysis indicated that *B. schroederi* had undergone earlier demographic expansion.

**Conclusions:**

These results confirmed that *B. schroederi* populations do not follow a pattern of isolation by distance, further revealing the possible existence of physical connections before geographic separation. This study should also contribute to an improved understanding of the population genetics and evolutionary biology of *B. schroederi* and assist in the control of baylisascariasis in giant pandas.

**Electronic supplementary material:**

The online version of this article (doi:10.1186/s13071-014-0606-3) contains supplementary material, which is available to authorized users.

## Background

Genetic markers are used increasingly to reconstitute the epidemiological history of human and veterinary diseases and to identify environmental factors underlying the spread of pathogens [1-6]. However, most of these studies focus on bacterial or viral diseases. For parasitic diseases, the complex life cycle of the causative organisms (such as helminthes), including different developmental stages and hosts, make their transmission heavily dependent on multiple environmental factors. Traditional approaches to study parasites, particularly parasitic nematodes, often concern their pathogenicity, prevalence and possible vaccines, but the complex evolutionary relationships of parasite populations are limitedly studied [7,8]. Recently, DNA sequence data have been successfully used to characterize and understand the genetic diversity of parasites and to explain and predict their likely dynamic importance. Moreover, such genetic techniques have been also applied to identify recurrent co-evolutionary units within larger systems and to study how parasites survive short-term environmental perturbations and evolve in response to long-term environmental changes [9]. Of course, a range of factors should be balanced when choosing suitable DNA markers, including cost and the purpose for which they are used. Mitochondrial DNA (mtDNA) sequences are powerful and reliable molecular markers for detecting population structures and inferring population differences as a result of its mutational rates which are proposed to be more rapid than nuclear DNA, and presumed lack of recombination and maternal inheritance. Additionally, mtDNA contains both rapidly diverging and highly conserved regions, thus making different mtDNA regions suitable for resolving distinct taxonomic issues in parasites [10-12]. For example, the genes for mitochondrial cytochrome c oxidase subunits I-III (*cox1-3*) and nicotinamide dehydrogenase subunit IV (*nad4*) have recently been used to investigate and compare the genetic variation and population structure of the parasitic nematodes *Baylisascaris columnaris* [13], *Dictyocaulus viviparous* [14], and *Haemonchus contortus* [15,16]. Moreover, Iñiguez et al. genetically characterized *Ascaris* spp. from humans and pigs using the mitochondrial cytochrome c oxidase subunit 1 (*cox1*) and NADH dehydrogenase subunit 1 (*nad1*) genes, and demonstrated that the haplotypes *cox1* H14P3 and *nad1* H12P17 are widely separated in Brazil [17].

The giant panda (*Ailuropoda melanoleuca*), a flagship species for wildlife conservation, is one of the world’s most recognized and threatened animals with a total wild population size estimated to be only 1,600; these individuals are restricted to six mountain ranges in China (Minshan, Qionglai, Qinling, Daxiangling, Xiaoxiangling and Liangshan), of which 44.36% are in Minshan, 27.38% in Qionglai and 17.23% in Qinling [18-20]. Infection with the parasitic nematode *Baylisascaris schroederi* (Ascaridida: Nematoda) is the most important cause of death in wild giant pandas and thus it poses a significant threat to these populations [21,22]. Adult stages of this parasite usually inhabit the intestines of the giant panda, while migrating larval stages can disseminate into various body tissues. Damage to bodily tissues can include extensive inflammation and scarring of the intestinal wall and parenchyma of the liver and lungs (caused by larvae), as well as intestinal obstruction, inflammation and even death (mainly caused by adults) [23-25]. As with other ascaridoids, *B. schroederi* infection follows ingestion and the life cycle is complete without the need for an intermediate host. Infective second-stage (L2) larvae hatch in the small intestine, migrate into the liver and the lung, and finally arrive at the small intestine where they mature, mate and produce eggs [23]. These eggs are highly resistant to decontamination and environmental degradation and can remain viable in moist soil for years, thus acting as a substantial reservoir for new infections. In nature, *B. schroederi* infection rate among wild pandas is often over 50% and can even be 100%, thus making it a leading cause of death in wild populations [21,22]. Despite its detrimental health impact on wild pandas, this parasite was first described only in 1939 as *Ascaris schroederi* [26], before being renamed as *B. schroederi* in 1968 [27]. Subsequent studies regarding *B. schroederi* focused mainly on morphological characteristics, fundamental biology, life cycle, pathogenicity, vaccine development and prevalence [22,23,28]*.* Recent advancements in PCR and sequencing technologies have led to the publication of several gene fragment sequences from *B. schroederi* (e.g., *ITS-1*, *ITS-2* and 5.8S), as well as complete mtDNA and microRNA [21,25,29,30]. These data have contributed to an improved understanding for the molecular biology and genetics of this parasite. However, studies to date that are involved to the genetic diversity of *B. schroederi* remain scarce, with only one report publically available in which a single mt *cytb* was utilized for this purpose [18].

Considering the limitation of using just a single gene to define the genetic structure of a parasite [31-33], the aim of this present study was to combine two different mtDNA markers, *cox1* and *atp6*, which possess a symmetrical evolutionary rate (a higher genetic variation of *atp6* than *cox1*), to expand and improve our knowledge of the genetic diversity of *B. schroederi.* Our data analysis included plotting transition and transversion frequencies of these genes and determining the genetic distances between populations. These data provide more detailed information into the genetic diversity of *B. schroederi* populations in three endemic areas of China (Minshan, Qionglai and Qinling mountain ranges), and enable a reevaluation of the population structure. In addition, we compared the genetic diversity of the population and inferred the phylogenetic relationships between different isolates according to their geographical distribution.

## Methods

### Specimen collection

**Figure 1:**
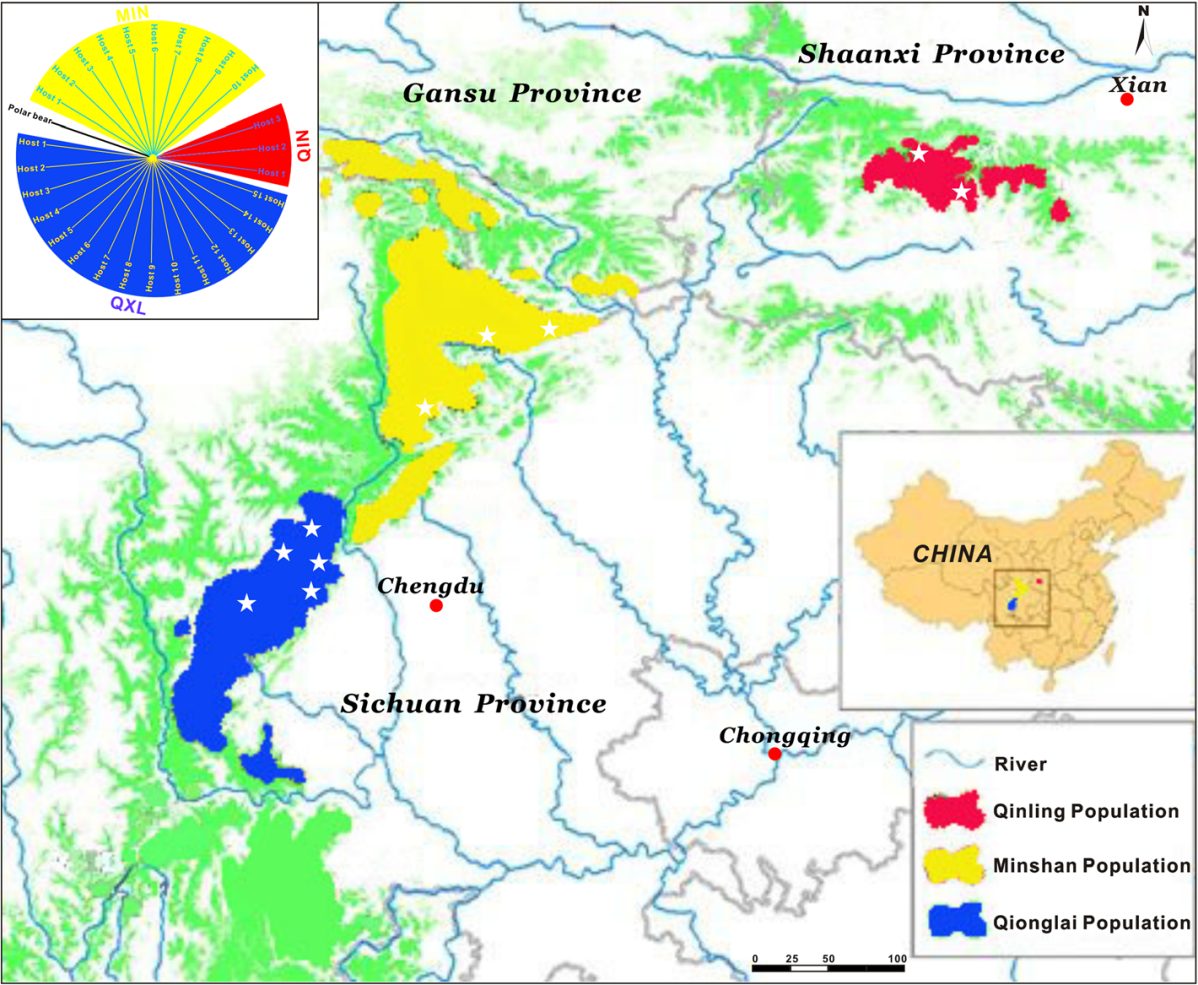
**Map of the sampling sites** ***.*** The geographical locations of *B. schroederi* isolates included in this study are marked with white stars, and the parasite specimens are from three different mountain range populations in China: Qinling (red), Minshan (yellow) and Qionglai (blue). Likewise, three giant panda populations (Qinling, red; Minshan, yellow; Qionglai-Liangshan-Daxiangling-Xiaoxiangling, blue) and their corresponding evolutionary relationships are shown in the upper left corner (for details, please see ref. [55]). This information is used to illustrate the possible co-evolution between the parasite and its host.

Between two and three parasite specimens were collected per giant panda (10-15 pandas per mountain range), except for Qinling (see Figure 1 and Additional file 1: Table S1 for geographical sampling details). Fifty-seven worms originating from 28 dead, injured or rescued giant pandas from three mountain ranges (Minshan, Qionglai and Qinling) were obtained during 1994-2014 and all were included in this present study. All worms were washed in physiological saline buffer, identified according to morphology [22], and stored at -20°C for preservation.

### Laboratory methods

Genomic DNA was extracted from 0.5 g of tissue from each individual *B. schroederi* by standard sodium dodecyl sulfate/proteinase K treatment and phenol/chloroform extraction, followed by column-purification using the Wizard® SV Genomic DNA Purification System (Promega, USA), eluted with 30 μL of elution buffer (pH 8.0) according to the manufacturers’ recommendations, and then stored at -20°C for PCR amplification. Two pairs of PCR primers were designed to amplify the complete mt *atp6* and *cox1* genes using Primer Premier 5.0 software (Premier Biosoft International, Palo Alto, CA, USA) and the published mtDNA sequence of *B. schroederi* (GenBank Accession No.: HQ671081). The primer pairs were as follows: *atp6*, forward: 5′-CGCGGATCCTTCGATATTCGTGGCCT-3′, and reverse: 5′-CGCAAGCTTCTAATATGGTGTCTTCGG-3′; *cox1*, forward: 5′-TTTAGAGGTTGGAATGTAGGGT-3′, and reverse: 5′- CCATCCCCTTAATCTGCAAT-3′. All PCR reactions contained ~20 ng of genomic DNA and were carried out in 50-μL reaction volumes containing 25 μL 2× Phusion High-Fidelity PCR Master Mix (Finnzymes OY, Espoo, Finland), 2 μL of each primer, 2 μL DNA and 19 μL of ddH_2_O under the following cycling conditions: an initial denaturation at 95°C for 5 min; then for *cox1*, 35 cycles at 95°C for 1 min, 50°C for 1 min, and 72°C for 1 min; but for *atp6*, 35 cycles of 95°C for 45 s, 50°C for 45 s, and 72°C for 45 s; followed by a final step at 72°C for 10 min. Negative controls were included in each PCR to test for possible contamination. All PCR products were examined on agarose gels (1%) to verify that they represented the target bands. The corrected gel-isolated fragments were subsequently column-purified and sequenced. Sequencing was performed by Invitrogen (Invitrogen Biotechnology Co. Ltd., Shanghai, China) using terminator-based cycle sequencing with BigDye chemistry (Applied Biosystems, Foster City, CA, USA) on an ABI 3730 DNA sequencer (Applied Biosystems). A “primer-walking” strategy (in both directions) was used and, to ensure maximum accuracy, each amplicon was sequenced twice independently. A third PCR product was sequenced in case of any discrepancies. Consensus sequences were deposited in the GenBank database.

### Phylogenetic and population genetic analyses

Sequences with consensus lengths (600 bp for *atp6* and 1578 bp for *cox1*) were aligned using the ClustalW program in MEGA v.5.0 [34]. Nucleotide alignments were generated based on protein alignments according to codon alignment. Phylogenetic relationships and nucleotide/haplotype diversity were initially determined for each mitochondrial gene. Given the lack of variable and parsimony-information sites for each gene, the sequence data from *atp6* and *cox1* were concatenated into a single 2,178-bp fragment for each specimen for realignment according to the aforementioned method. During this procedure, any ambiguous regions within these alignments were filtered with Gblocks 0.91 b [35]. Phylogenetic trees inferred from the *atp6*, *cox1* and pooled datasets were constructed using the maximum parsimony (MP) method, with each node estimated using 1,000 bootstrap replicates. Data were plotted with MEGA v.5.0 [34]. To further define the current population structure of *B. schroederi*, a Bayesian statistical method [36], coupled with the best-fit evolutionary model GTR + I + G determined by jModeltest [37] using the Akaike Information Criterion (AIC), was also used to analyze the genetic variation among populations based on the two mitochondrial genes separately and in combination (the model GTR + I + G was determined for both *atp6* and *cox1*, and was directly applied in the following concatenated data). A total of 10 million generations were run and the trees were sampled every 10,000 generations. When the mean standard deviation of the split frequencies had reduced to less than 0.01, the first 250 trees were discarded as “burn-in” and the remaining trees were used to generate a 50% majority rule consensus tree for each data set. Homologous sequences obtained from *Baylisascaris transfuga* (Accession No. NC-015924.1 for both mitochondrial *atp6* and *cox1* genes) were used as outgroups in all phylogenetic analyses due to the close relationship between *B. schroederi* and *B. transfuga* [21]*.* In addition, the Data Analysis in Molecular Biology and Evolution (DAMBE) program [38] was used to establish plots of transition (s) and transversion (v) frequencies against the K80 distance, and then the amino acid substitution propensity of these two genes was estimated.

### Population differentiation

Aligned FASTA files were collapsed into variable sites and haplotypes for parsimony network reconstruction using Dna SP 5.0 [39]. A statistical parsimony network to infer relationships among sequences that had recently diverged was created with Network 4.6 [40]. Haplotype (Hd) and nucleotide diversity (π) were calculated by Dna SP 5.0 [39]. Furthermore, a Mantel test was conducted to calculate the correlation between pairwise geographies using Arlequin version 3.0 [41]. Based on the same program, the coefficient of gene differentiation (*G*st) and index of genetic differentiation (*F*st) were also estimated, and the corresponding gene flows (*N*m) between populations were indirectly calculated as follows: *N*m = 0.5*(1-*G*st)/*G*st [42] or *N*m = (1-*F*st)/4*F*st [43]. Of these parameters, the *G*st statistic has been used widely to evaluate population subdivision on the basis of the divergence of allele frequencies from random mating [44]. Finally, to evaluate whether the genetic differentiation between populations was associated with the geographical isolation of the mountains, analysis of molecular variance (AMOVA) [41] was performed in Arlequin. The resulting data was used to examine population genetic structure and variation within and between the three sampling locations.

### Historical demography of *B. schroederi* populations

Different approaches were used to infer historical demographic expansions, including those of Fu’s Fs, Tajima’s D and the mismatch-distribution tests [45-47]. To assess whether population expansion occurred in *B. schroederi*, 1,000 simulations in Arlequin [41] were performed to obtain the distribution of Fu’s Fs and Tajima’s D values under neutrality and to obtain *P* values for each population. Moreover, to measure frequency distributions of pairwise differences between sample sequences, mismatch-distribution analysis was also performed using Dna SP 5.0 [39] for 1,000 simulations.

### Ethics statement

This study was reviewed and approved by the Research Ethics Committee in College of Veterinary Medicine, Sichuan Agricultural University (Ya’an, China; Approval No. 2011-028). All parasite samples were collected from giant pandas after the permissions of the Forestry Department of Sichuan Province and Shaanxi Rare Wildlife Rescue Breeding Research Center, with no specific permits being required by the authority for the parasite collection.

## Results

### Haplotype sequence analysis

**Figure 2:**
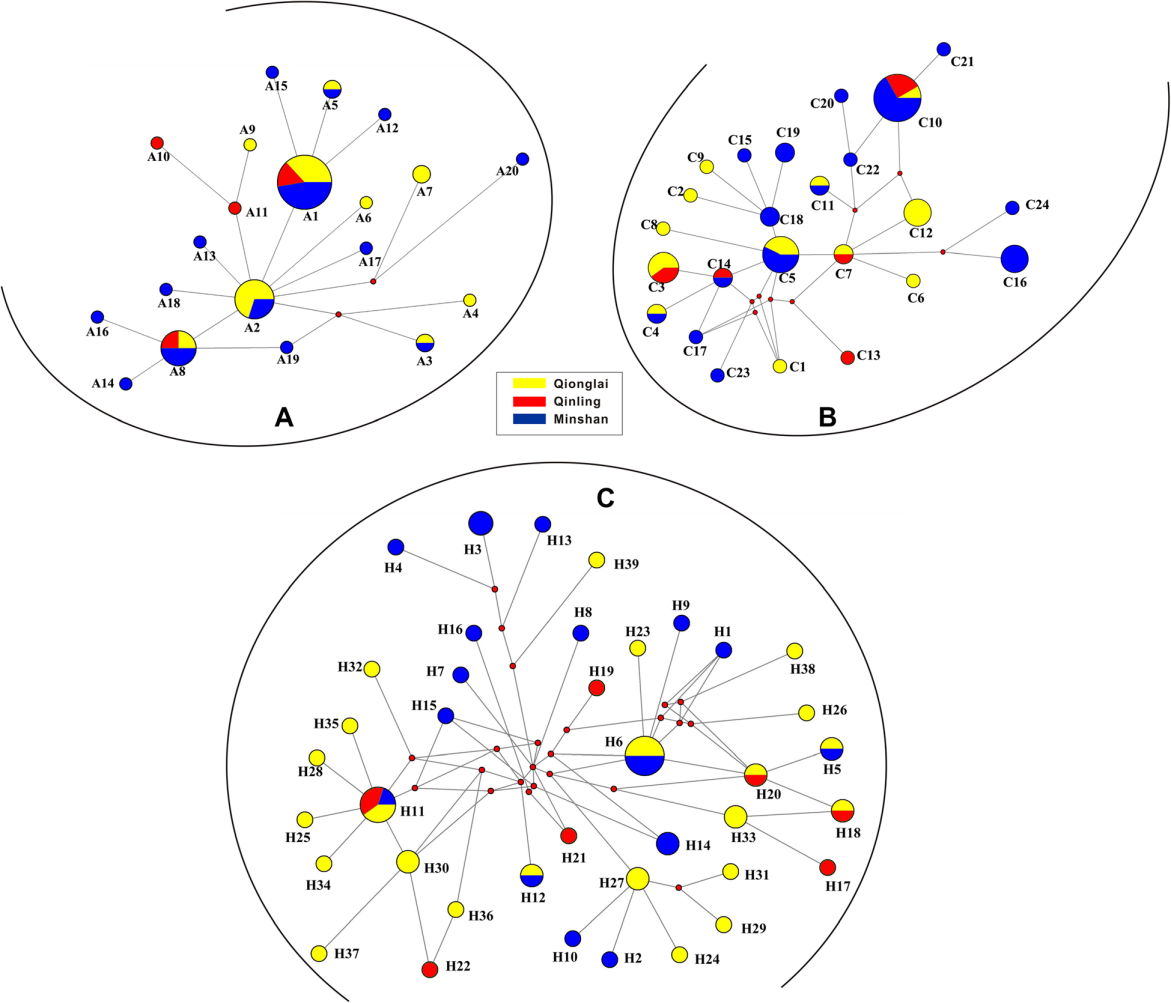
**Network maps of** ***atp6*** **(A),** ***cox1*** **(B) and the concatenated sequence (C) haplotypes of** ***B. schroederi.*** The area of each circle is proportional to the haplotype frequency.

The 600-bp *atp6* and 1,578-bp *cox1* sequences from 57 *B. schroederi* isolates were deposited in GenBank under accession numbers KJ587749-KJ587805 and KJ587806-KJ587862, respectively. No insertion/deletions (indels) were detected. In total, 20 haplotypes (A1-20) were identified in *atp6*, 24 haplotypes (C1-24) in *cox1* and 39 haplotypes (H1-39) in *atp6* + *cox1*. Haplotypes A1 and A8 (inferred from *atp6*), C10 (inferred from *cox1*) and H11 (inferred from concatenated sequences) were shared by all samples collected from the three mountain ranges (Figure 2). Furthermore, A1, C10 and H11 haplotypes were used as alignment references to discover further variants. Interestingly, each mountain range population appeared to be associated with its own set of haplotypes. For example, the haplotypes C1, C2, C5, C6, C8, C9 and C12 deduced from *cox1* were the typical haplotypes of the *B. schroederi* population in Qionglai. However, of the 39 haplotypes inferred from the concatenated sequences, just six haplotypes were shared by two or three of the mountain range populations (Figure 2C). Additionally, the genetic diversity of the 57 *B. schroederi* isolates from the three different regions was calculated (see Tables 1, 2, 3 and 4). A high level of haplotype diversity was maintained in the *B. schroederi* populations, but their nucleotide diversity was relatively low due to the richness of single-nucleotide substitutions. Of these populations, the Minshan mountain range population exhibited greatest haplotype diversity (0.84795 for *atp6* and 0.92982 for *cox1*) and nucleotide diversity (0.00359 for *atp6* and 0.00204 for *cox1*). However, the concatenated sequences seemed to have greater degree of haplotype diversity (all values >0.9) when compared with *atp6* and *cox1* (Table 4). In addition, nucleotide and deduced amino acid sequences were aligned. The maximum Kimura distance between the three *B. schroederi* populations was 0.003 for both *atp6* and *cox1*. However, the estimated transition/transversion bias (*R*) differed between *atp6* and *cox1*, and the *R*-value between the three populations was lower for *cox1* (22.10) than for of *atp6* (50.48) (data not shown).

**Table 1:** **Comparison of** ***atp6*** **and** ***cox1*** **genetic differentiation between the three** ***B. schroederi*** **populations**

**Population 1**	**Population 2**	***atp6*** **gene**				***cox1*** **gene**			
		**Coefficient of gene differentiation**		**Index of genetic differentiation**		**Coefficient of gene differentiation**		**Index of genetic differentiation**	
		***N*** **m**	***G*** **st**	***N*** **m**	***F*** **st**	***N*** **m**	***G*** **st**	***N*** **m**	***F*** **st**
Minshan	Qinling	98.74	0.00253	18.79	0.01349	9.59	0.02541	9.14	0.02669
Minshan	Qionglai	−39.82	−0.00632	23.67	0.01068	10.27	0.02377	11.29	0.02170
Qionglai	Qinling	11.51	0.02126	−23.70	−0.01066	13.13	0.01869	−8.83	−0.02913

**Table 2:** **Summary of the genetic diversity of the three populations of** ***B. schroederi*** **collected from giant pandas inhabiting different mountain ranges according to the** ***cox1*** **gene**

**Populations**	**No. of individuals**	**No. of haplotypes**	**No. of variable sites**	**Haplotype diversity**	**Nucleotide diversity**	**Tajima’s D**	**Fu’s Fs**
Minshan	19	12	18	0.92982	0.00204	−1.43052*	−5.164
Qinling	8	5	10	0.85714	0.00260	0.32140	0.373
Qionglai	30	15	18	0.90345	0.00268	−0.23778*	−4.339*
Total	57	24	29	0.92794	0.00268	−1.20886*	−11.065**

*P < 0.05; **P < 0.01; ***P < 0.001.

**Table 3:** **Summary of the genetic diversity of the three populations of** ***B. schroederi*** **collected from giant pandas inhabiting different mountain ranges according to the** ***atp6*** **gene**

**Populations**	**No. of individuals**	**No. of haplotypes**	**No. of variable sites**	**Haplotype diversity**	**Nucleotide diversity**	**Tajima’s D**	**Fu’s Fs**
Minshan	19	9	13	0.84795	0.00359	−1.54920*	−3.188*
Qinling	8	4	5	0.75000	0.00321	0.00046	0.081
Qionglai	30	14	18	0.87356	0.00330	−1.94387*	−8.521*
Total	57	20	27	0.84524	0.00336	−2.11978*	−13.735**

*P < 0.05; **P < 0.01; ***P < 0.001.

**Table 4:** **Summary of the genetic diversity of the three populations of** ***B. schroederi*** **collected from giant pandas inhabiting different mountain ranges according to the concatenated** ***atp6*** **and** ***cox1*** **sequences**

**Populations**	**No. of individuals**	**No. of haplotypes**	**No. of variable sites**	**Haplotype diversity**	**Nucleotide diversity**	**Tajima’s D**	**Fu’s Fs**
Minshan	19	16	34	0.97661	0.00274	−1.54549*	−7.842*
Qinling	8	7	15	0.96429	0.00277	0.22163	−1.480
Qionglai	30	24	37	0.98391	0.00270	−1.35598*	−15.344*
Total	57	39	56	0.97870	0.00274	−1.73588*	−29.912**

*P < 0.05; **P < 0.01; ***P < 0.001.

### Phylogenetic analysis

**Figure 3:**
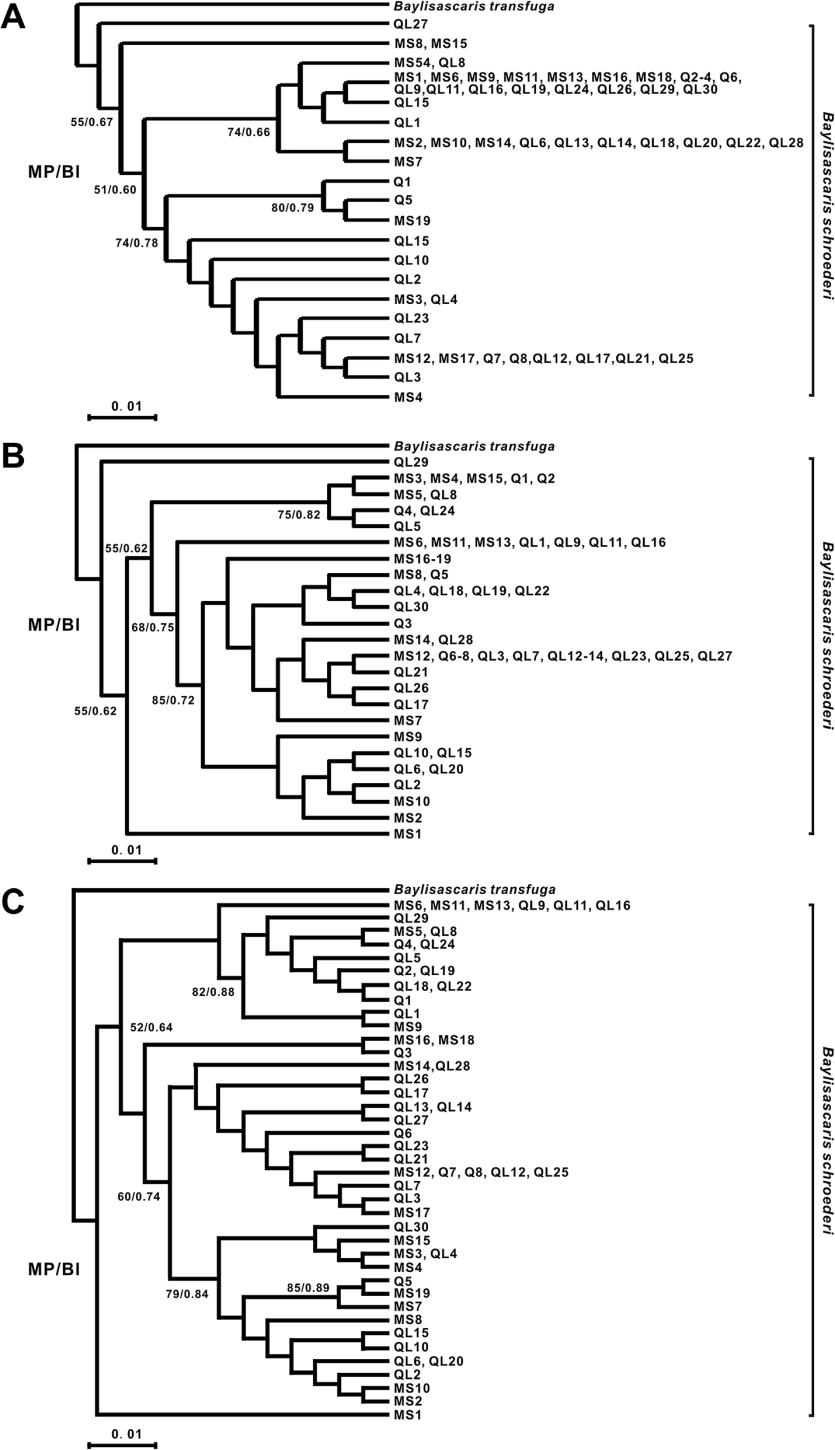
**Maximum parsimony (MP) and Bayesian inference (BI) trees for the 57** ***atp6*** **,** ***cox1*** **and concatenated gene sequences.** The numbers along the branches indicate bootstrap values from different analyses in the order: MP/BI. *B. transfuga* was used as the outgroup. **A** depicts *atp6* sequences; **B** depicts *cox1* sequences; and **C** depicts the concatenated sequences.

To study the phylogenetic relationships between the *B. schroederi* isolates collected from different geographic regions, the *atp6* and *cox1* genes were analyzed, separately or in combination, to infer the phylogeny, and the corresponding constructed phylogenetic trees are shown in Figure 3. The samples in the three trees clustered into a mixed group with low posterior probability values, indicating that they would be difficult to distinguish from each other by phylogenetic analyses due to the small differences existing between individuals isolated from the different ranges. Therefore, it was impossible to elucidate evolutionary events based only on such undefined phylogenetic trees, and this meant that further network analysis was required.

### Network analysis

In addition to phylogenetic trees, an entire network for all haplotypes was produced (Figure 2). Based on the 24 *cox1* haplotypes, the network map revealed a star-like pattern around haplotype C5, which contained seven individuals, with three in the Qionglai and four in the Minshan populations. A total of 12 other haplotypes were found in the Qionglai population (C1–12), while there were 5 haplotypes present in the Qinling population (C3, C7, C10, C13 and 14). Interestingly, the haplotype C10 was shared by samples derived from all three mountain ranges, while haplotypes C4, C5 and C11 were shared by only the Qionglai and Minshan populations and C14 by only the Minshan and Qinling populations (Figure 2B, Additional file 2: Table S2). The remaining haplotypes appeared to be highly dispersed, and no obvious correlations were observed between sample clusters. For *atp6*, although the network analysis showed some patterns consistent with those revealed by *cox1*, lower haplotype resolution was observed when compared with *cox1* (Figure 2A). Of these, haplotype A2, which originated from Qionglai and Minshan, was surrounded by the other haplotypes, but haplotype A1 was found in more individuals (19/57) than A2 (10/57). Within the concatenated sequence data, a more complex network was observed when compared with that produced for *atp6* or *cox1* genes alone*.* Further, 37 of the 39 haplotypes appeared to be around H11 and H6 haplotypes and formed two separate star-like patterns (Figure 2C). Of note, the haplotype H11 was surrounded by most of the isolates derived from the Qionglai population. To investigate the information of these sequences further, the number of haplotypes in each population was also calculated (see Additional file 2: Table S2).

### Population genetic structure

Based on population genetic structure analysis, the genetic diversity of *cox1* between the different *B. schroederi* isolates was highly consistent with that of *atp6*, but slightly lower than that of *atp6* and *cox1* in combination (Table 2, 3, 4 and data not shown). Interestingly, the corresponding nucleotide diversity (π) based on either *atp6*, *cox1* or *atp6* + *cox1* exhibited significant stability between *B. schroederi* populations. In addition, for *atp6*, the genetic differentiation within populations was significant (*P* < 0.05) for all pairwise comparisons, which was in agreement with results from the phylogeny and network analyses. A similar result was also found for the concatenated sequence data (Table 4). In the *cox1* data, 97.81% of the genetic variation was attributed to differences between individuals within populations, while the remaining 2.19% was due to differences between *B. schroederi* populations. These results confirmed that there was lower genetic differentiation between than within populations across the three *B. schroederi* populations examined here. This conclusion was validated further by the high rates of gene flow determined between different *B. schroederi* populations (Table 1). To test whether new *B. schroederi* isolates would constitute separate populations, *G*st and *F*st statistics were calculated to evaluate the variation in allele frequencies between *B. schroederi* populations. *G*st values varied from 0 to 1 with values > 0.25 and *F*st values ranged from 0 to 1 with values >0.27. As shown in Table 1, the highest *G*st and *F*st values for *cox1* were observed between Minshan and Qinling populations (*G*st = 0.02541, *P* > 0.05; *F*st = 0.02669, *P* > 0.05), while the lowest *G*st and *F*st values were found between Qinling and Qionglai populations (*G*st = 0.01869, *P* > 0.05; *F*st = -0.02913, *P* > 0.05). Interestingly, a similar trend was also observed for *F*st values of *atp6*, but this was contrasted with *G*st values of *atp6*, where the highest *G*st value was detected between the Qinling and Qionglai populations (*G*st = 0.02126, *P* >0.05) (Table 1).

### Population expansion

**Figure 4:**
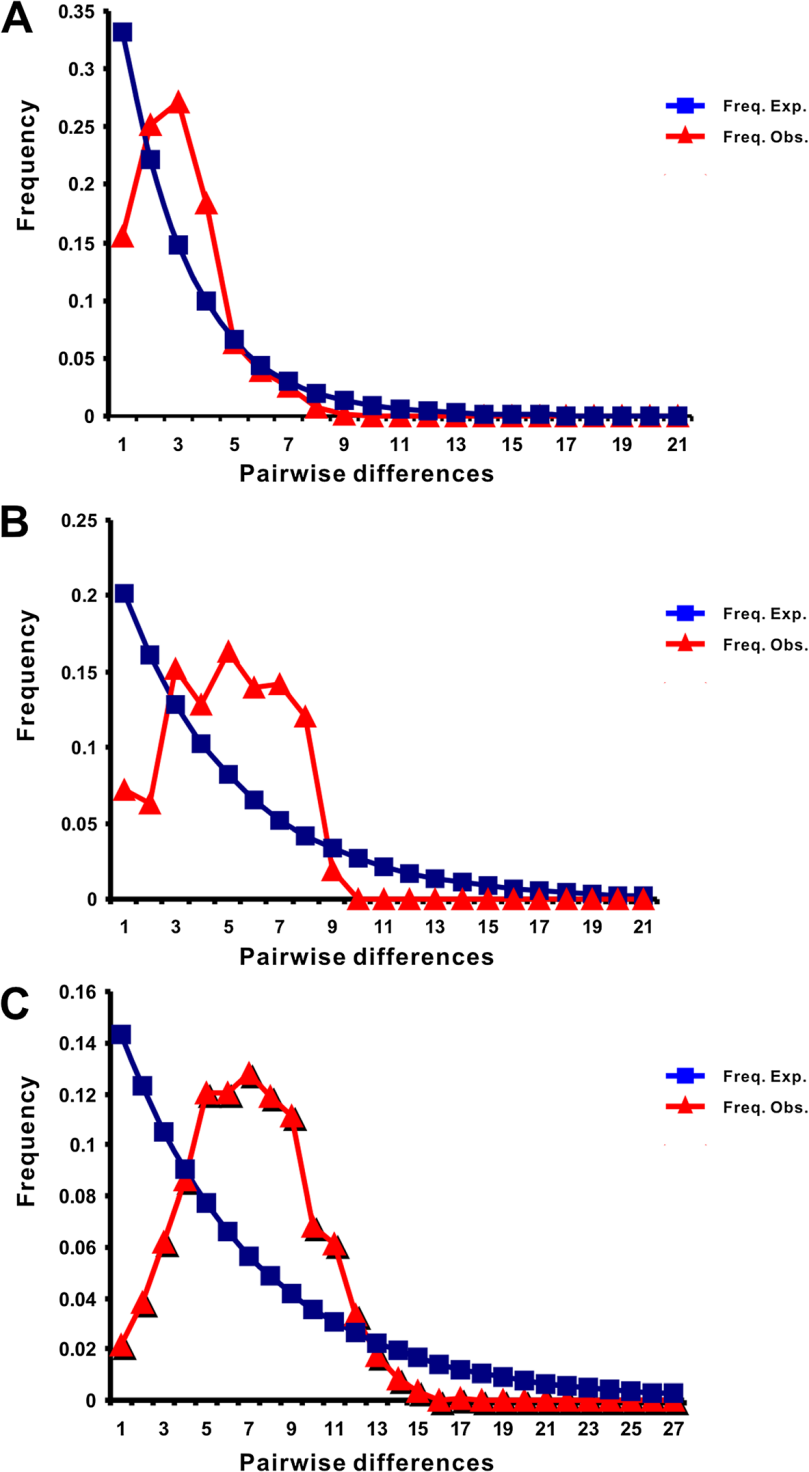
**Mismatch-distribution to test the expansion of** ***atp6*** **(A),** ***cox1*** **(B) and concatenated sequences (C) in the population of 57** ***B. schroederi*** **isolates.** The number of nucleotide differences between pairs of sequences is indicated by the x-axis, while their frequency is indicated by the y-axis.

Based on *cox1*, *atp6* and concatenated sequence datasets, Fu’s Fs and Tajima’s D statistics as well as the mismatch-distribution test were used separately to explore the demographic histories of the three *B. schroederi* populations. Of these, the results of the mismatch analysis inferred from either *atp6* or concatenated data provided consistent insights into the demographic history of *B. schroederi*: all three populations (Minshan, Qionglai and Qinling) were unimodal and had undergone at least one expansion event (Figure 4A and 4C). Although the mismatch-distribution of the *cox1* gene presented a multimodal outcome (Figure 4B), the statistical parameters RG and SSDs (not shown) meant that the hypothesis of population expansion could not be rejected. Additionally, the results of Fu’s Fs and Tajima’s D tests under neutrality generated by *atp6*, *cox1* and concatenated sequences gave negative values (-13.735 for *atp6*, -11.065 for *cox1* and -29.912 for *atp6* + *cox1* in Fu’s Fs, respectively; -2.11978 for *atp6*, -1.20886 for *cox1* and -1.73588 for *atp6* + *cox1* in Tajima’s D, respectively) (Table 2, 3 and 4). Thus, these findings confirmed the existence of population expansion in the demographic history of all three *B. schroederi* populations and further supported the conclusions drawn from the mismatch analysis.

## Discussion

Parasites are a crucial component of natural communities, since they can directly or indirectly alter the structure of the communities by impacting the number of free-living species or their relative abundance [48]. With advances in molecular genetics, rapidly increased knowledge concerning the genetic diversity of nematodes is becoming a prerequisite to elucidate basic biological and population characteristics of these parasites. More importantly, a better understanding of parasitic nematode population dynamics is fundamental to design new strategies to monitor and control these problematic organisms. Excitingly, many recent studies have used genetic markers (e.g., mitochondrial and nuclear DNA) to depict geographical movements of parasitic nematodes [49-51]. MtDNA markers are receiving increased attention for this purpose due to higher *F*st values than observed for nuclear DNA counterparts [31-33,52,53]. However, given the different evolutionary rates occurring in different regions of nematode mtDNAs [54] and the high degree of conservation of the *cytb* gene [18], in this present study two mitochondrial markers (*atp6* and *cox1*) were combined to further explore the genetic variation and population structure of *B. schroederi*.

In this study, low *G*st and *F*st values indicated small genetic differentiation in the *B. schroederi* population (Table 1), and this was further supported by AMOVA analysis. Likewise, other factors also confirmed a non-significant level of genetic differentiation of the *B. schroederi* populations. For example, a high rate of gene flow (*N*m > 9) between the three populations was always observed for both *atp6* and *cox1*, and it could be speculated that the effective number of *B. schroederi* individuals that exchanged genetic material was sufficient to overcome the effects of genetic drift. Thus, *B. schroederi* isolated from different geographical regions may be a homogenous species with strong population diffusion, little obstruction for gene flow, simple genetic structure and small differentiation. Moreover, the parasite is a specialized organism that parasitizes a specific host, and the genetics and behavior of the host can also have an important impact on parasite genomic variation. The population history of the giant panda, as characterized by Zhao et al. (2012), showed that pandas in the Qinling mountain range were distinct from other populations (including Minshan and Qionglai-Liangshan-Daxiangling-Xiaoxiangling) due to geographical isolation, human activities and global changes in climate [55]. However, contrary to our expectations, *B. schroederi* showed only weak population subdivisions (Figure 2), particularly the Qinling population that shared frequent gene flow with the Minshan population. To a certain extent, these results implied that the evolutionary rate of *B. schroederi* may be disharmonious with its host. Similar conjectures have been proposed to interpret evolutionary relationships between the parasitic nematodes *Syphacia obvelata* and *Trichuris muris* and their host *Mus musculus* [56]. Additionally, previous studies have indicated that a clear genetic structure would arise when inbreeding occurred, whereas outcrossing species tended to show little genetic structure [57,58]. Therefore, we speculated that *B. schroederi* could be an ‘outcrossing’ species. This hypothesis is supported by knowledge of the genetic and reproductive behavior of the parasite and its host (for details, please see refs. [22,23,59]): (i) *B. schroederi* is a dioecious parasitic nematode with a direct developmental lifecycle; (ii) *B. schroederi* can be transmitted from one geographical origin to another because of the cross-regional reproductive behavior of pandas; (iii) thus, mating of *B. schroederi* could occur between individuals from different geographical areas. Alternatively, there could be another reason for possible mixed infections in the hosts, as *B. schroederi* specimens from the same individuals had low genetic differences and different haplotypes. Perhaps future complete mitochondrial genome-based population analysis involving a larger *B. schroederi* population would provide novel and/or further insights into this issue.

The other important finding of this present study was the low level of genetic diversity found across all three *B. schroederi* populations, which was similar to a previous report [18]. Low nucleotide diversity was observed in *B. schroederi* and this revealed a relative lack of genetic variation across the ascaridoid, regardless of geographical origin and population size. Interestingly, similar findings were also described for other *Ascaris* species, including *Ascaris galli* [50], *Ascaris suum* [60,61] and *Parascaris equorum* [51]. Together, these results implied that low level of diversity may be a common feature of ascaridoid nematodes. In addition, the leptokurtic distribution of the haplotypes showed several frequent haplotypes (C5, C10, C14; A1, A2, A8; H6, H11) as well as numerous rare haplotypes (Figure 2). These results indicated that the patterns of genetic differentiation were similar between the two mitochondrial genes (*atp6* and *cox1*), which was probably due to the small sample size; however, the genetic diversity in the Qinling population was less clear. The analysis of *atp6* and *cox1* genes showed low geographic separation between the three mountain range populations, which was consistent with the findings of a previous report [18]. Furthermore, network maps showed that the three populations only shared haplotypes A1 and A8 for *atp6*, C10 for *cox1* and H11 for *atp6* + *cox1*. The most frequent haplotypes were A1 of *atp6*, C10 of *cox1* and H11 of *atp6* + *cox1* in the examined populations (Additional file 2: Table S2). This finding indicated that A1, C10 and H11 might be the most ancient haplotypes, as these often displayed a high frequency and tended towards a widespread geographic distribution [62]. Of note, the haplotype A2 of *atp6* was surrounded by nine clades while haplotype A1 was surrounded by just four, and this same phenomenon was observed for haplotypes C5 of *cox1* and H6 of *atp6* + *cox1* (C5 and H6 had more clades than C10 and H11). Interestingly, haplotypes A2, C5 and H6 were shared only by Minshan and Qionglai mountain range populations. Thus, it could be speculated that these three haplotypes differentiated after from the ancient haplotype and would display a trend of widespread geographic distribution and may evolve to form a single haplotype across all three mountain ranges in the future. Taken together, our data indicated that the *B. schroederi* species was not genetically differentiated.

In general, certain aspects of parasites often closely track counterparts of their host’s biology due to long-term interplay between them [63,64]. In the *B. schroederi*-panda co-evolutionary system, the host has recently experienced two population expansions, two bottlenecks and two divergences [18]. Thus, it is reasonable to assume that the demographic history of the panda may influence that of its specialized parasite *B. schroederi*. Encouragingly, negative Fu’s Fs (high) and Tajima’s D (low) values (Table 2, 3 and 4) and the results of the mismatch analysis (Figure 4) confirmed that *B. schroederi* had been subjected to a sudden demographic expansion in the past [65], and this was consistent with the hypothesis of Zhou et al. (2013). Furthermore, across the three habitats of giant pandas examined, the prevalence and intensity of *B. schroederi* infections is relatively high (50–100%) [21,22]. No commercial vaccine is available against *B. schroederi*, and its control relies largely on anthelmintic drugs. However, some studies have reported the occurrence of drug resistance in *B. schroederi* populations in China [23]. Given high levels of gene flow among *B. schroederi* populations and the potential of rare resistance alleles to spread, an investigation of drug resistance in *B. schroederi* is urgently needed.

## Conclusion

This investigation initially combined two different mitochondrial markers, *atp6* and *cox1*, to explore population genetic variability and structure of 57 *B. schroederi* isolates sampled from the three main giant panda habitats in China. The low levels of genetic diversity and high levels of gene flow indicated that *B. schroederi* was not genetically differentiated and had experienced lineage re-arrangement. Moreover, the negative Fu’s Fs and Tajima’s D values coupled with mismatch analysis consistently showed that the *B. schroederi* population could have been subjected to a sudden demographic expansion in the past. These results, together with previous studies, further suggest that *B. schroederi* populations did not follow a pattern of isolation by distance, revealing the possible existence of physical connections before the populations became geographically separated. This finding should contribute to a deeper understanding of population genetics and evolutionary biology of *B. schroederi*, a nematode parasite of the giant panda.

## Additional files

Additional file 1: Table S1. Origin and number of *B. schroederi* isolates and their hosts in China. Additional file 2: Table S2a. Detailed haplotype information of the *cox1* gene. **Table S2b:** Detailed haplotype information of the *atp6* gene. **Table S2c:** Detailed haplotype information of the *atp6* + *cox1* genes).

## Abbreviations

mtDNA: Mitochondrial DNA
*atp6*: ATPase subunit 6
*cox1*: Cytochrome c oxidase subunit I
*cox3*: Cytochrome c oxidase subunit III
*nad4*: Nicotinamide dehydrogenase subunit IV
L2: Second-stage
AMOVA: Analysis of molecular variance
MP: Maximum parsimony
